# Supplementary material for: Ecology of the collapse of Rapa Nui society
Source: Proc Biol Sci. 2020 Jun 24;287(1929):20200662. doi: 10.1098/rspb.2020.0662 (PMC7329031; doi:10.1098/rspb.2020.0662)
Supplement: Basic data sets and R scripts of the analyses [file rspb20200662supp1.docx]

Electronic Supplementary Material

**Table S1**. Description list of the estimated parameters of the population models fitted to the data.

| **Parameter** | **Definition** |
| --- | --- |
| N_t_ | Population abundance (N) in time t |
| N_t-1_ | Population abundance (N) in time t-1 |
| r_N_ | Maximum per capita growth rate |
| K | A constant that accounts for competition intensity and resource depletion represented by the availability of crop land and productivity (i.e. equilibrium population size, carrying capacity) |
| F_t-1_ | Palm forest cover (Pollen %) at time t-1 |
| K(F_t-1_) | The parameter K as a function of the palm forest cover |
| C_t-1_ | Southern oscillation index (SOI) at time t-1 |
| K(C_t-1_) | The parameter K as a function of SOI |
| K(F_t-1_,C_t-1_) | The parameter K as a function of palm cover forest and SOI both at time t-1 |
| α | Intercept in a lineal model of pollen % of palm tree as a function of N and C |
| β | Coefficient of N in a lineal model fro pollen % of palm tree |
| γ | Coefficient of C in a lineal model fro pollen % of palm tree |
| ω | Coefficient of the interaction of N and C in a lineal model fro pollen % of palm tree |
| ε | Error term in a lineal model fro pollen % of palm tree |

**Table S2**. **Population dynamic models for the human population at Rapa Nui.**

Parameter values are given in the equations. Best models were selected considering the Akaike Information Criteria for small sample size (*AIC_c_)*. Model parameters were estimated by nonlinear regression analysis in R-program using the nls library. The model notations are: *N*, Summed Probabilities Densities (SPD) as a proxy of population size; F is the pollen % of palm trees in the records of lake Raraku [20], C is the reconstructed Southern Oscillation Index [38] averaged for periods of 30 yr; *p*, number of model parameters; ∆AIC_c_ = model AIC_c_ – lowest AIC_c_; w_i_, Akaike weights, *R^2^* is the coefficient of prediction of the simulated dynamics.

| Models | Loglik | AIC | AIC_c_ | ΔAIC_c_ | *w* | *p* | *R^2^* |
| --- | --- | --- | --- | --- | --- | --- | --- |
| Model for human dynamics |  |  |  |  |  |  |  |
| $1. N_{t}= N_{t-1}\cdot e^{\left[ 0.47\cdot\left( 1-\frac{N_{t-1}}{0.0017} \right) \right]}$ | 11.42 | -16.83 | -14.48 | 31.97 | 0.00 | 3 | 0.65 |
| $2. N_{t}= N_{t-1}\cdot e^{\left[ 0.50\cdot\left( 1-\frac{N_{t-1}}{\left( {6.6\cdot10}^{-4}+7.7\cdot{10}^{-5}\cdot F_{t-1} \right)} \right) \right]}$ | 26.19 | -44.29 | -40.62 | 5.83 | 0.05 | 4 | 0.94 |
| $3. N_{t}= N_{t-1}\cdot e^{\left[ 0.50\cdot\left( 1-\frac{N_{t-1}}{\left( 0.004-0.0007\cdot C_{t-1} \right)} \right) \right]}$ | 26.11 | -44.11 | -40.47 | 5.98 | 0.05 | 4 | 0.94 |
| $4. N_{t}= N_{t-1}\cdot e^{\left[ 0.51\cdot\left( 1-\frac{N_{t-1}}{\left( 0.0025+4.2\cdot{10}^{-5}\cdot F_{t-1}-4.3\cdot{10}^{-4}\cdot C_{t-1} \right)} \right) \right]}$ | 31.03 | -51.84 | -46.45 | 0.00 | 0.91 | 5 | 0.94 |

**Table S3**. **Lineal regression model for the change in pollen% of palms.** Where *F* = pollen%, *N* = is the (SPD) the Summed Probabilities Densities, and *C* = the average reconstructed Southern Oscillation Index (SOI) [38]. Best model (in bold face) was selected considering the Akaike Information Criteria for small sample size (*AIC_c_)*.

| **Models** | **Loglik.** | **AIC_c_** | **ΔAIC_c_** | ***w*** | ***w_i_/w_j_*** | ***p*** | ***r^2^*** |
| --- | --- | --- | --- | --- | --- | --- | --- |
| $\boldsymbol{1. F= 46.54- 19357\cdot N}$ | **-62.09** | **132.37** | **0.00** | **0.58** | **1.00** | **3** | **0.44** |
| $2. F= 28.10- 13.15\cdot C$ | -64.28 | 136.74 | 4.37 | 0.07 | 8.91 | 3 | 0.25 |
| $3. F= 43.20- 15578\cdot N-6.07\cdot C$ | -61.39 | 134.79 | 2.42 | 0.17 | 3.36 | 4 | 0.45 |
| $4. F= 43.73- 15374\cdot N-4.62\cdot C-1961.\cdot N\cdot C$ | -61.37 | 139.42 | 7.05 | 0.02 | 33.95 | 5 | 0.40 |
| $5. F= 30.46-8.14\cdot C-6369.3\cdot N\cdot C$ | -64.14 | 140.27 | 7.90 | 0.01 | 52.01 | 4 | 0.20 |
| $6. F= 45.69- 15850\cdot N-6220\cdot N\cdot C$ | -61.51 | 135.02 | 2.65 | 0.15 | 3.77 | 4 | 0.44 |

**Table S4:** ^14^C dates used for reconstructing paleodemographic trends in Rapa Nui over the past 1200 years. “Marine” indicates the proportion of marine carbon from bioanthropological data according to [28].

| **Site** | **Laboratory ID** | **Age**  **(^14^C years BP)** | **SD**  **(^14^C years BP)** | **pmarine** | **Material** | **Source** |
| --- | --- | --- | --- | --- | --- | --- |
| Ahu Nau Nau plaza | AA-27343 | 371 | 50 | 1 | Algal nodule | [27] |
| Ahu Nau Nau plaza | AA-27344 | 423 | 60 | 1 | Algal nodule | [27] |
| Ahu Nau Nau plaza | AA-27346 | 430 | 40 | 1 | Algal nodule | [27] |
| Ahu Nau Nau plaza | AA-27345 | 555 | 45 | 1 | Algal nodule | [27] |
| Ahu Akahanga | UCIAMS-105150 | 225 | 15 | 0.355 | Colagen human tooth | [28] |
| Ahu Akahanga | UCIAMS-105153 | 235 | 15 | 0.311 | Colagen human tooth | [28] |
| Ahu Akahanga | UCIAMS-105159 | 295 | 15 | 0.362 | Colagen human tooth | [28] |
| Ahu Kihi Kihi Rau Mea | UCIAMS-105151 | 175 | 15 | 0.312 | Colagen human tooth | [28] |
| Ahu Kihi Kihi Rau Mea | UCIAMS-105154 | 155 | 15 | 0.373 | Colagen human tooth | [28] |
| Ahu Kihi Kihi Rau Mea | UCIAMS-105149 | 150 | 15 | 0.375 | Colagen human tooth | [28] |
| Ahu Kihi Kihi Rau Mea | UCIAMS-105162 | 195 | 15 | 0.291 | Colagen human tooth | [28] |
| Ahu Mahatua | UCIAMS-105148 | 185 | 15 | 0.5 | Colagen human tooth | [28] |
| Ahu Nau Nau | UCIAMS-105152 | 160 | 25 | 0.309 | Colagen human tooth | [28] |
| Ahu Nau Nau | UCIAMS-105158 | 605 | 15 | 0.305 | Colagen human tooth | [28] |
| Ahu Nau Nau | UCIAMS-105163 | 400 | 15 | 0.239 | Colagen human tooth | [28] |
| Ahu Nau Nau | UCIAMS-105281 | 390 | 45 | 0.323 | Colagen human tooth | [28] |
| Ahu One Makihi | UCIAMS-105155 | 235 | 15 | 0.454 | Colagen human tooth | [28] |
| Ahu Tongariki | UCIAMS-105156 | 295 | 15 | 0.399 | Colagen human tooth | [28] |
| Ahu Tongariki | UCIAMS-105144 | 440 | 15 | 0.56 | Colagen human tooth | [28] |
| Ahu Tongariki | UCIAMS-105145 | 550 | 15 | 0.34 | Colagen human tooth | [28] |
| Ahu Tongariki | UCIAMS-105146 | 270 | 20 | 0.439 | Colagen human tooth | [28] |
| Ahu Tongariki | UCIAMS-105147 | 345 | 20 | 0.446 | Colagen human tooth | [28] |
| Ahu Tongariki | UCIAMS-105160 | 195 | 15 | 0.426 | Colagen human tooth | [28] |
| Ahu Tongariki | UCIAMS-105161 | 645 | 15 | 0.274 | Colagen human tooth | [28] |
| Ahu Tongariki | UCIAMS-105277 | 370 | 25 | 0.535 | Colagen human tooth | [28] |
| Ahu Tongariki | UCIAMS-105278 | 125 | 30 | 0.369 | Colagen human tooth | [28] |
| Ahu Tongariki | UCIAMS-105279 | 175 | 30 | 0.35 | Colagen human tooth | [28] |
| Ahu Tongariki | UCIAMS-105280 | 310 | 15 | 0.496 | Colagen human tooth | [28] |
| Ahu Tongariki | UCIAMS-105282 | 465 | 15 | 0.368 | Colagen human tooth | [28] |
| Ana Oroi | UCIAMS-105157 | 185 | 15 | 0.343 | Colagen human tooth | [28] |
| Ahu Ature Huki | T-7979 | 510 | 80 | 0 | Wood charcoal | [27] |
| Ahu Ature Huki | Ua-1144 | 580 | 85 | 0 | Wood charcoal | [27] |
| Ahu Heki´i | Ua-11700 | 705 | 45 | 0 | Carbonized nutshell | [27] |
| Ahu Heki´i | Ua-11702 | 465 | 45 | 0 | Carbonized nutshell | [27] |
| Ahu Heki´i | Ua-11701 | 700 | 45 | 0 | Carbonized nutshell | [27] |
| Ahu Heki´i | Ua-11703 | 555 | 50 | 0 | Carbonized nutshell | [27] |
| Ahu Ihu Arero | Gak-4616 | 480 | 90 | 0 | Wood charcoal | [27] |
| Ahu Ihu Arero | Gak-4617 | 480 | 90 | 0 | Wood charcoal | [27] |
| Ahu Motu Toremo Hiva | KIA-29813 | 610 | 25 | 0 | Wood charcoal | [27] |
| Ahu Motu Toremo Hiva | KIA-29812 | 630 | 25 | 0 | Wood charcoal | [27] |
| Ahu Motu Toremo Hiva | KIA-26453 | 675 | 25 | 0 | Wood charcoal | [27] |
| Ahu Motu Toremo Hiva | KIA-26464 | 700 | 25 | 0 | Wood charcoal | [27] |
| Ahu Motu Toremo Hiva | KIA-26461 | 630 | 25 | 0 | Wood charcoal | [27] |
| Ahu Motu Toremo Hiva | KIA-26452 | 675 | 20 | 0 | Wood charcoal | [27] |
| Ahu Motu Toremo Hiva | KIA-26487 | 240 | 20 | 0 | Wood charcoal | [27] |
| Ahu Motu Toremo Hiva | KIA-29814 | 325 | 25 | 0 | Wood charcoal | [27] |
| Ahu Motu Toremo Hiva | KIA-26483 | 150 | 20 | 0 | Wood charcoal | [27] |
| Ahu Nau Nau I | Ua-34183 | 535 | 35 | 0 | Carbonized nutshell | [27] |
| Ahu Nau Nau I | T-7342 | 710 | 70 | 0 | Wood charcoal | [27] |
| Ahu Nau Nau II | T-7347 | 720 | 120 | 0 | Wood charcoal | [27] |
| Ahu Nau Nau III | Ua-617 | 610 | 85 | 0 | Wood charcoal | [27] |
| Ahu Nau Nau IV? | T-7348 | 200 | 80 | 0 | Wood charcoal | [27] |
| Ahu No. 31-286 | Ua-11704 | 795 | 50 | 0 | Wood charcoal | [27] |
| Ahu o Rongo | GrA-18378 | 655 | 30 | 0 | Wood charcoal | [27] |
| Ahu o Rongo I | GrA-18380 | 655 | 30 | 0 | Wood charcoal | [27] |
| Ahu o Rongo I | GrN-26318 | 715 | 35 | 0 | Wood charcoal | [27] |
| Ahu o Tuki | Beta-155733 | 595 | 65 | 0 | Carbonized nutshell | [27] |
| Ahu o Tuki | Beta-155732 | 610 | 60 | 0 | Carbonized nutshell | [27] |
| Ahu o Tuki | GrA-25870 | 640 | 35 | 0 | Carbonized nutshell | [27] |
| Ahu Ra´ai | Ua-13163 | 135 | 60 | 0 | Wood charcoal | [27] |
| Ahu Ra´ai | Ua-13164 | 515 | 60 | 0 | Wood charcoal | [27] |
| Ahu Ra´ai | Ua-13165 | 570 | 50 | 0 | Wood charcoal | [27] |
| Ahu Ra´ai | Ua-13166 | 635 | 50 | 0 | Wood charcoal | [27] |
| Ahu Ra´ai | Ua-13167 | 645 | 50 | 0 | Wood charcoal | [27] |
| Ahu Tahai | Gak-4507 | 200 | 70 | 0 | Wood charcoal | [27] |
| Ahu Tautira | Ua-13161 | 220 | 50 | 0 | Wood charcoal | [27] |
| Ahu Tautira | Ua-13284 | 475 | 60 | 0 | Wood charcoal | [27] |
| Ahu Tautira | Ua-13162 | 720 | 50 | 0 | Wood charcoal | [27] |
| Ahu Vai Teka | M-1372 | 330 | 100 | 0 | Wood charcoal | [27] |
| Ahu Vai Teka | I-455 | 340 | 75 | 0 | Wood charcoal | [27] |
| Ahu Vai Teka | TBN-348-2 | 399 | 76 | 0 | Wood charcoal | [27] |
| Ahu Vinapu 1 | K-523 | 440 | 100 | 0 | Wood charcoal | [27] |
| Ahu Vinapu 2 | Ua-19464 | 605 | 45 | 0 | Carbonized nutshell | [27] |
| Ahu Vinapu 2 | Ua-19463 | 610 | 40 | 0 | Carbonized nutshell | [27] |
| Ahu Vinapu 2 | M-710 | 1100 | 200 | 0 | Wood charcoal | [27] |
| Akahanga settlement | Beta-099353 | 200 | 50 | 0 | Wood charcoal | [27] |
| Akahanga settlement | Beta-099334 | 320 | 60 | 0 | Wood charcoal | [27] |
| Akahanga settlement | Beta-099343 | 220 | 70 | 0 | Wood charcoal | [27] |
| Akahanga settlement | Beta-099333 | 340 | 60 | 0 | Wood charcoal | [27] |
| Akahanga settlement | Beta-099330 | 220 | 70 | 0 | Wood charcoal | [27] |
| Akahanga settlement | Beta-099354 | 390 | 110 | 0 | Wood charcoal | [27] |
| Ana Kai Tangata | Beta-38785 | 290 | 80 | 0 | Wood charcoal | [27] |
| Anakena 35-8 | UGa-630 | 395 | 75 | 0 | Wood charcoal | [27] |
| Anakena cultural layer | Ua-34189 | 565 | 35 | 0 | Carbonized nutshell | [27] |
| Anakena cultural layer | Beta-47172 | 170 | 110 | 0 | Wood charcoal | [27] |
| Anakena cultural layer | T-7977 | 220 | 80 | 0 | Wood charcoal | [27] |
| Anakena cultural layer | T-7958 | 340 | 100 | 0 | Wood charcoal | [27] |
| Anakena cultural layer | T-6680 | 370 | 90 | 0 | Wood charcoal | [27] |
| Anakena cultural layer | T-7959 | 510 | 40 | 0 | Wood charcoal | [27] |
| Anakena cultural layer | T-7974 | 540 | 60 | 0 | Wood charcoal | [27] |
| Anakena cultural layer | T-7349 | 550 | 150 | 0 | Wood charcoal | [27] |
| Anakena cultural layer | T-7344 | 600 | 140 | 0 | Wood charcoal | [27] |
| Anakena cultural layer | Beta-47171 | 660 | 80 | 0 | Wood charcoal | [27] |
| Anakena cultural layer | T-7975 | 710 | 40 | 0 | Wood charcoal | [27] |
| Anakena cultural layer | T-7350 | 710 | 80 | 0 | Wood charcoal | [27] |
| Anakena cultural layer | T-7343 | 750 | 100 | 0 | Wood charcoal | [27] |
| Anakena cultural layer | T-7976 | 789 | 90 | 0 | Wood charcoal | [27] |
| Anakena cultural layer | T-7345 | 810 | 80 | 0 | Wood charcoal | [27] |
| Anakena cultural layer | T-7346 | 810 | 70 | 0 | Wood charcoal | [27] |
| Anakena cultural layer | Beta-47173 | 860 | 100 | 0 | Wood charcoal | [27] |
| Anakena cultural layer | T-7341 | 900 | 120 | 0 | Wood charcoal | [27] |
| Anakena cultural layer | Beta-47169 | 900 | 80 | 0 | Wood charcoal | [27] |
| Anakena cultural layer | Beta-47170 | 900 | 60 | 0 | Wood charcoal | [27] |
| Anakena cultural layer | T-6679 | 1170 | 140 | 0 | Wood charcoal | [27] |
| Anakena Dune Site | Beta-196714 | 590 | 60 | 0 | Wood charcoal | [27] |
| Anakena Dune Site | Beta-196711 | 660 | 40 | 0 | Wood charcoal | [27] |
| Anakena Dune Site | Beta-196713 | 670 | 60 | 0 | Wood charcoal | [27] |
| Anakena Dune Site | Beta-196712 | 680 | 60 | 0 | Wood charcoal | [27] |
| Anakena Dune Site | Beta-196715 | 710 | 40 | 0 | Wood charcoal | [27] |
| Anakena Dune Site | Beta-196716 | 720 | 60 | 0 | Wood charcoal | [27] |
| Anakena Dune Site | Beta-209903 | 870 | 80 | 0 | Wood charcoal | [27] |
| Anakena Dune Site | Beta-209904 | 870 | 40 | 0 | Wood charcoal | [27] |
| Anakena E2 | K-522 | 430 | 100 | 0 | Wood charcoal | [27] |
| Anakena pre-Ahu Nau Nau I | Ua-34188 | 665 | 30 | 0 | Wood charcoal | [27] |
| Anakena pre-Ahu Nau Nau I | Ua-34186 | 555 | 35 | 0 | Wood charcoal | [27] |
| Anakena settlement | Ua-34190 | 665 | 35 | 0 | Wood charcoal | [27] |
| Anakena settlement | Ua-34191 | 565 | 35 | 0 | Carbonized nutshell | [27] |
| CS2 | Beta-144308 | 740 | 40 | 0 | Wood charcoal | [27] |
| CS2 | Beta-144309 | 250 | 40 | 0 | Wood charcoal | [27] |
| CS4 | Beta-144306 | 790 | 80 | 0 | Wood charcoal | [27] |
| CS4 | Beta-144307 | 840 | 40 | 0 | Wood charcoal | [27] |
| East Poike | KIA-18838 | 588 | 22 | 0 | Wood charcoal | [27] |
| East Poike | KIA-18837 | 631 | 22 | 0 | Wood charcoal | [27] |
| East Poike | KIA-20383 | 317 | 20 | 0 | Carbonized nutshell | [27] |
| East Poike | SRR-2430 | 820 | 40 | 0 | Carbonized nutshell e rat-gnawed | [27] |
| Hanga te Pahu | CNRS 1111b | 465 | 85 | 0 | Carbonized nutshell | [27] |
| Hanga te Pahu | CNRS 1111a | 640 | 90 | 0 | Carbonized nutshell | [27] |
| Maunga Orito | Beta-178860 | 200 | 50 | 0 | Wood charcoal | [27] |
| Maunga Orito | KIA-17117 | 148 | 18 | 0 | Wood charcoal | [27] |
| Maunga Orito | KIA-17116 | 177 | 18 | 0 | Wood charcoal | [27] |
| Maunga Orito | KIA-25975 | 347 | 21 | 0 | Wood charcoal | [27] |
| Maunga Orito | Beta-196925 | 450 | 40 | 0 | Wood charcoal | [27] |
| Maunga Orito | Beta-196926 | 460 | 40 | 0 | Wood charcoal | [27] |
| Moai Tukuturi | T-5006 | 180 | 40 | 0 | Wood charcoal | [27] |
| Moai Tukuturi | T-6258 | 230 | 60 | 0 | Wood charcoal | [27] |
| Moai Tukuturi | Beta-13130 | 540 | 90 | 0 | Wood charcoal | [27] |
| Moai Tukuturi | Ua-618 | 1040 | 90 | 0 | Wood charcoal | [27] |
| Orongo | T-193 | 540 | 70 | 0 | Wood charcoal | [27] |
| Orongo | Beta-099336 | 320 | 70 | 0 | Wood charcoal | [27] |
| Orongo | Beta-099342 | 240 | 60 | 0 | Wood charcoal | [27] |
| Orongo | Beta-099356 | 200 | 50 | 0 | Wood charcoal | [27] |
| Orongo | Beta-099347 | 210 | 50 | 0 | Wood charcoal | [27] |
| Orongo | Beta-099339 | 250 | 50 | 0 | Wood charcoal | [27] |
| Orongo | K-506 | 220 | 100 | 0 | Wood charcoal | [27] |
| Orongo | K-514 | 380 | 60 | 0 | Wood charcoal | [27] |
| Orongo | T-194 | 470 | 70 | 0 | Wood charcoal | [27] |
| Orongo | K-520 | 540 | 100 | 0 | Wood charcoal | [27] |
| Poike Ditch | Beta-208937 | 440 | 60 | 0 | Wood charcoal | [27] |
| Poike Ditch | Beta-208938 | 190 | 40 | 0 | Wood charcoal | [27] |
| Poike Ditch | Beta-47281 | 760 | 80 | 0 | Wood charcoal | [27] |
| Rock Garden 16, Hiva Hiva Lava Flow | Beta-238061 | 300 | 40 | 0 | Wood charcoal | [27] |
| Rock Garden 20, Hiva Hiva Lava Flow | Beta-238062 | 570 | 40 | 0 | Wood charcoal | [27] |
| Rock Garden 22, Hiva Hiva Lava Flow | Beta-237464 | 560 | 40 | 0 | Wood charcoal | [27] |
| Rock Garden 25, Hiva Hiva Lava Flow | Beta-238063 | 210 | 40 | 0 | Wood charcoal | [27] |
| Site 1-187 | WSU-1146 | 1180 | 230 | 0 | Wood charcoal | [27] |
| Site 10-241 | Beta-47365 | 380 | 60 | 0 | Wood charcoal | [27] |
| Site 10-241 | Beta-47366 | 480 | 60 | 0 | Wood charcoal | [27] |
| Site 10-241 | Beta-47367 | 390 | 50 | 0 | Wood charcoal | [27] |
| Site 14-1 | UGa-631 | 395 | 60 | 0 | Wood charcoal | [27] |
| Site 15-233 | Beta-254389 | 160 | 40 | 0 | Wood charcoal | [27] |
| Site 15-233 | Beta-254390 | 280 | 40 | 0 | Wood charcoal | [27] |
| Site 15-233 | Beta-237461 | 150 | 40 | 0 | Wood charcoal | [27] |
| Site 15-233 | Beta-237462 | 670 | 40 | 0 | Wood charcoal | [27] |
| Site 15-68 | Beta-237460 | 440 | 40 | 0 | Wood charcoal | [27] |
| Site 15-68 | Beta-237459 | 840 | 40 | 0 | Wood charcoal | [27] |
| Site 15-90 | Beta-237457 | 460 | 40 | 0 | Wood charcoal | [27] |
| Site 18-228 | Beta-47282 | 400 | 60 | 0 | Wood charcoal | [27] |
| Site 18-419 | Beta-47283 | 330 | 60 | 0 | Wood charcoal | [27] |
| Site 18-473G | Beta-199322 | 220 | 40 | 0 | Wood charcoal | [27] |
| Site 18-473G | Beta-199324 | 1110 | 40 | 0 | Wood charcoal | [27] |
| Site 18-473G | Beta-199323 | 570 | 50 | 0 | Twig and grass fragments | [27] |
| Site 20-52 | Beta-144310 | 780 | 50 | 0 | Wood charcoal | [27] |
| Site 20-52 | Beta-144311 | 380 | 40 | 0 | Wood charcoal | [27] |
| Site 21-568a, Hare Aio | Beta-47279 | 320 | 70 | 0 | Wood charcoal | [27] |
| Site 21-568a, Hare Aio | Beta-46110 | 430 | 70 | 0 | Wood charcoal | [27] |
| Site 26-1, Ahu Te Niu (Ahu Ohau) | AZ-22 | 325 | 40 | 0 | Carbonized nutshell | [27] |
| Site 26-1, Ahu Te Niu (Ahu Ohau) | AZ-28 | 555 | 40 | 0 | Carbonized nutshell | [27] |
| Site 26-1, Ahu Te Niu (Ahu Ohau) | AZ-14 | 230 | 60 | 0 | Wood charcoal | [27] |
| Site 26-1, Ahu Te Niu (Ahu Ohau) | B-95879 | 230 | 90 | 0 | Wood charcoal | [27] |
| Site 26-1, Ahu Te Niu (Ahu Ohau) | AZ-8 | 375 | 45 | 0 | Wood charcoal | [27] |
| Site 26-1, Ahu Te Niu (Ahu Ohau) | AZ-13 | 535 | 50 | 0 | Wood charcoal | [27] |
| Site 26-1, Ahu Te Niu (Ahu Ohau) | AZ-24 | 535 | 40 | 0 | Wood charcoal | [27] |
| Site 26-1, Ahu Te Niu (Ahu Ohau) | B-106319 | 570 | 50 | 0 | Wood charcoal | [27] |
| Site 26-1, Ahu Te Niu (Ahu Ohau) | AZ-25 | 650 | 40 | 0 | Wood charcoal | [27] |
| Site 26-1, Ahu Te Niu (Ahu Ohau) | AZ-23 | 685 | 50 | 0 | Wood charcoal | [27] |
| Site 26-1, Ahu Te Niu (Ahu Ohau) | B-95878 | 700 | 90 | 0 | Wood charcoal | [27] |
| Site 26-22, Te Niu | AZ-2 | 605 | 40 | 0 | Wood charcoal | [27] |
| Site 26-22, Te Niu | AZ-3 | 385 | 40 | 0 | Wood charcoal | [27] |
| Site 26-36, Te Niu | AZ-4 | 210 | 60 | 0 | Wood charcoal | [27] |
| Site 26-50, Te Niu | B-95880 | 550 | 110 | 0 | Wood charcoal | [27] |
| Site 26-6, Te Niu | AZ-9 | 230 | 45 | 0 | Wood charcoal | [27] |
| Site 26-6, Te Niu | AZ-7 | 680 | 45 | 0 | Wood charcoal | [27] |
| Site 26-7b, Te Niu | AZ-1 | 435 | 40 | 0 | Wood charcoal | [27] |
| Site 26-I0, Te Niu | AZ-5 | 715 | 70 | 0 | Wood charcoal | [27] |
| Site 26-T0, Te Niu | AZ-6 | 415 | 70 | 0 | Wood charcoal | [27] |
| Site 26F, Te Niu | AZ-27 | 545 | 40 | 0 | Wood charcoal | [27] |
| Site 31-90 | Beta-099355 | 150 | 50 | 0 | Wood charcoal | [27] |
| Site 31-90 | Beta-099346 | 160 | 50 | 0 | Wood charcoal | [27] |
| Site 31-90 | Beta-099345 | 610 | 80 | 0 | Wood charcoal | [27] |
| Site 31-90 | Beta-91771 | 420 | 80 | 0 | Burnt grass | [27] |
| Site 31-98 | Beta-91772 | 130 | 40 | 0 | Wood charcoal | [27] |
| Site 31-98 | Beta-91774 | 130 | 60 | 0 | Wood charcoal | [27] |
| Site 31-98 | Beta-91775 | 240 | 40 | 0 | Wood charcoal | [27] |
| Site 32-200c | Gd-6963 | 150 | 90 | 0 | Organic material | [27] |
| Site 35-11 | Beta-28753 | 280 | 60 | 0 | Wood charcoal | [27] |
| Site 7-1, Akahanga cave | I-7517 | 220 | 80 | 0 | Wood charcoal | [27] |
| Site 7-1, Runga Vae cave | I-7515 | 190 | 80 | 0 | Wood charcoal | [27] |
| Site 7-553 | Beta-50136 | 270 | 60 | 0 | Wood charcoal | [27] |
| Site 7-553 | Beta-50138 | 440 | 60 | 0 | Wood charcoal | [27] |
| Site 8-85 | SI-5460 | 580 | 60 | 0 | Wood charcoal | [27] |
| Site B, Inside a hare paenga near ahu | Beta-210553 | 640 | 40 | 0 | Wood charcoal | [27] |
| Site C, Hare umu, Ahu Te Pa Haha Tea | Beta-210555 | 210 | 40 | 0 | Wood charcoal | [27] |
| Site D, Ahu OPepe, inside corner of an L-shaped wall | Beta-210554 | 200 | 40 | 0 | Wood charcoal | [27] |
| ST 12 | Wk-24284 | 214 | 30 | 0 | Wood charcoal | [27] |
| ST 14 | Wk-27377 | 407 | 30 | 0 | Wood charcoal | [27] |
| ST 16 | Wk-24285 | 314 | 30 | 0 | Wood charcoal | [27] |
| ST 21 | Wk-27378 | 172 | 30 | 0 | Wood charcoal | [27] |
| ST 22 | Wk-24286 | 157 | 28 | 0 | Wood charcoal | [27] |
| ST 3 | Wk-27375 | 352 | 30 | 0 | Wood charcoal | [27] |
| ST 39 | Wk-27379 | 133 | 30 | 0 | Wood charcoal | [27] |
| ST 43 | Wk-27380 | 131 | 30 | 0 | Wood charcoal | [27] |
| ST 43 | Wk-27381 | 426 | 30 | 0 | Wood charcoal | [27] |
| ST 46 | Wk-27383 | 212 | 30 | 0 | Wood charcoal | [27] |
| ST 46 | Wk-27382 | 243 | 30 | 0 | Wood charcoal | [27] |
| ST 48 | Wk-27384 | 269 | 30 | 0 | Wood charcoal | [27] |
| ST 6 | Wk-27376 | 146 | 30 | 0 | Wood charcoal | [27] |
| Statue No. 478 | Ua-1145 | 180 | 110 | 0 | Wood charcoal | [27] |
| Statue Quarry | K-507 | 480 | 100 | 0 | Wood charcoal | [27] |
| Statue Quarry | Ua-14189 | 550 | 70 | 0 | Wood charcoal | [27] |
| SW Poike | KIA-19369 | 525 | 26 | 0 | Burnt grass | [27] |
| SW Poike | KIA-18834 | 319 | 21 | 0 | Wood charcoal | [27] |
| SW Poike | KIA-18833 | 482 | 26 | 0 | Wood charcoal | [27] |
| SW Poike | KIA-18832 | 570 | 22 | 0 | Wood charcoal | [27] |
| SW Poike | KIA-17110 | 654 | 22 | 0 | Wood charcoal | [27] |
| SW Poike | KIA-18841 | 297 | 21 | 0 | Wood charcoal | [27] |
| SW Poike | KIA-18842 | 298 | 48 | 0 | Wood charcoal | [27] |
| SW Poike | KIA-18839 | 561 | 26 | 0 | Wood charcoal | [27] |
| T 01, Stone-lined basin | Erl-13249 | 307 | 39 | 0 | Carbonized nutshell | [27] |
| T 01, Stone-lined basin | Erl-13248 | 360 | 39 | 0 | Carbonized nutshell | [27] |
| T 01, Stone-lined basin | Erl-13247 | 384 | 40 | 0 | Soil with organic remains | [27] |
| T 01, Stone-lined basin | Erl-13250 | 349 | 40 | 0 | Wood charcoal | [27] |
| Te Niu | AZ-26 | 240 | 40 | 0 | Wood charcoal | [27] |
| Test unit 107, Hiva Hiva Lava Flow | Beta-238582 | 490 | 40 | 0 | Carbonized nutshell | [27] |
| Viri o Tuki | GrA-25872 | 410 | 35 | 0 | Wood charcoal | [27] |

**Table S5:** Raw data used to implement population dynamic models for Rapa Nui. Percentage of palm pollen from the Raraku lacustrine record (12). SOI index: reconstructed Southern Oscillation Index by [38].

| **Calibrated age (CE)** | **Percentge of palm pollen** | **SOI index** |
| --- | --- | --- |
| 1892 | 9.48 |  |
| 1880 |  | -0.404 |
| 1850 |  | 0.744 |
| 1831 | 6.38 |  |
| 1820 |  | 0.356 |
| 1790 |  | 0.589 |
| 1769 | 6.98 |  |
| 1760 |  | 0.848 |
| 1730 |  | 1.495 |
| 1714 | 7.77 |  |
| 1700 |  | 1.645 |
| 1670 |  | 1.536 |
| 1640 |  | 1.068 |
| 1610 |  | 0.684 |
| 1580 |  | 1.225 |
| 1550 |  | 0.883 |
| 1528 | 7.55 |  |
| 1520 |  | 1.313 |
| 1490 |  | 0.681 |
| 1489 | 13.21 |  |
| 1460 |  | 0.086 |
| 1430 |  | 0.306 |
| 1442 | 27.49 |  |
| 1400 |  | 0.423 |
| 1392 | 16.75 |  |
| 1370 |  | -0.108 |
| 1349 | 25.00 |  |
| 1340 |  | -0.308 |
| 1310 |  | -0.497 |
| 1304 | 23.33 |  |
| 1280 |  | -0.696 |
| 1259 | 30.09 |  |
| 1250 |  | -1.128 |
| 1229 | 37.62 |  |
| 1220 |  | -1.605 |
| 1190 |  | -1.401 |
| 1168 | 64.22 |  |
| 1160 |  | -0.530 |
| 1130 |  | -0.034 |
| 1100 |  | -0.224 |
| 500 | 64.94 |  |
| 159 | 65.85 |  |
| -428 | 67.80 |  |
| -953 | 89.10 |  |
| -1292 | 91.47 |  |
| -1795 | 91.85 |  |

**Table S6:** Raw data used to fit population dynamic models (equation 4) for Rapa Nui. SPD = summed probability densities, R = per capita growth rates, Percentage of palm pollen from the Raraku lacustrine record was linearly interpolated [20]. SOI index: reconstructed Southern Oscillation Index by [38] averaged in a 30-yr time steps.

| **Calibrated age (CE)** | **SPD** | **R** | **Percentge of palm pollen** | **SOI index** |
| --- | --- | --- | --- | --- |
| 1130 | 0.00008 | 0.37000 | 64.90 | -0.287 |
| 1160 | 0.00012 | 0.50852 | 64.20 | -0.931 |
| 1190 | 0.00020 | 0.53208 | 37.60 | -1.583 |
| 1220 | 0.00035 | 0.42002 | 33.50 | -1.482 |
| 1250 | 0.00053 | 0.51779 | 30.08 | -0.947 |
| 1280 | 0.00088 | 0.40587 | 23.33 | -0.442 |
| 1310 | 0.00132 | 0.20326 | 25.00 | -0.441 |
| 1340 | 0.00162 | 0.16876 | 16.75 | -0.220 |
| 1370 | 0.00192 | 0.12759 | 22.00 | 0.062 |
| 1400 | 0.00218 | 0.04400 | 27.49 | 0.460 |
| 1430 | 0.00228 | -0.02958 | 20.00 | 0.201 |
| 1460 | 0.00221 | -0.19179 | 13.20 | 0.326 |
| 1490 | 0.00183 | -0.17038 | 10.40 | 1.029 |
| 1520 | 0.00154 | -0.06180 | 7.55 | 1.069 |
| 1550 | 0.00145 | -0.00043 | 7.40 | 0.985 |
| 1580 | 0.00145 | 0.01613 | 7.60 | 1.079 |
| 1610 | 0.00147 | -0.00820 | 7.60 | 0.738 |
| 1640 | 0.00146 | -0.12530 | 7.60 | 1.296 |
| 1670 | 0.00129 | -0.15345 | 7.60 | 1.632 |
| 1700 | 0.00110 | -0.06085 | 7.60 | 1.706 |
| 1730 | 0.00104 | 0.06900 | 7.60 | 1.258 |
| 1760 | 0.00111 | -0.00743 | 7.00 | 0.634 |

**Figure Captions ESM**


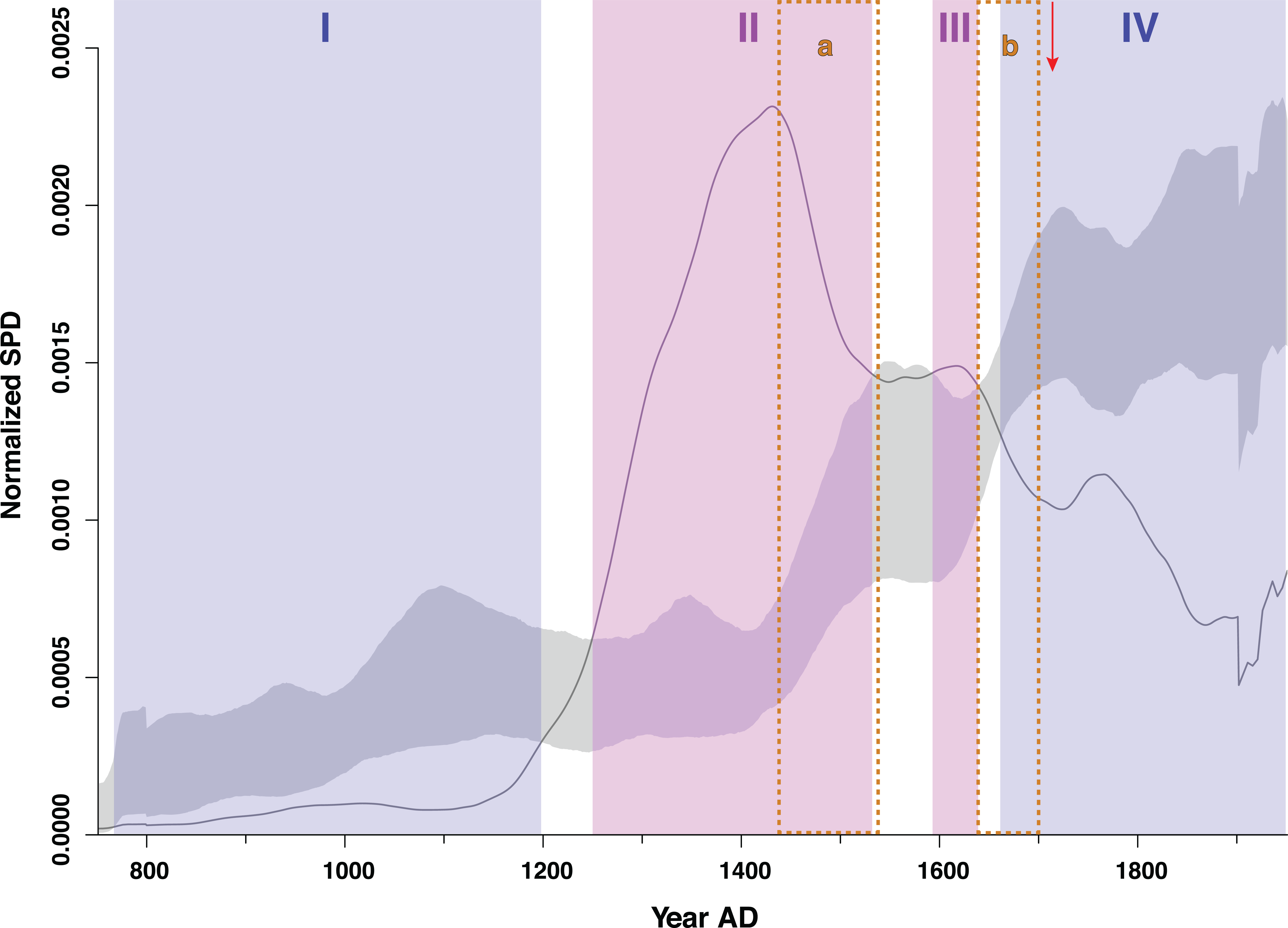


**Fig. S1.** Statistical hypothesis test for the Summed Probability Distribution (SPD) of archeological calibrated ^14^C-dates from Rapa Nui. The solid dark curve represents the normalized and smoothed (100-year rolling mean) SPD obtained by aggregating dates from the same site into 50-years bins. The grey envelope describes the 95% confidence band for the fitted lineal null model. Magenta and blue vertical bars indicate periods in that occur significant positive (i.e. genuine population rises) and negative (i.e. population falls) deviations at a global p-value of 0.0001, where I: 765- 11970 CE, II: 1250-1530 CE, III: 1590-1640 CE and IV: from 1660 CE onwards. Orange dashed bars denote population collapses reconstructed in this present study at 1430-1550 CE (a) and 1640-1700 CE (b) before the European contact (red arrow).

**R-scripts**

**#Fitting logistic models using the command nls for the time series of the summed probabilities density (SPD) of human data from Rapa Nui**

## Read the data

dat1<-read.table('RAPANUINW.txt',dec='.',sep='\t',header=TRUE)

## In the table Rt is the per capita growth rate of SPD data (proxy of human population), SPDt1 is the SPD value at time t-1 (human population at time t-1), Palm is % pollen of palm trees, and

## SOI is the SOI index plus a constant 2.6 to facilitate fitting (by eliminating negative values).

# 1.- Fitting the basic logistic model

modRNSPD1<-nls(Rt ~ b * (1 - (SPDt1/k)), data=dat1, start=list(b=0.47, k=0.002), trace=TRUE)

### Extracting parameter values

summary(modRNSPD1)

# 2.- Fitting the logistic model with palm cover as a lateral perturbation effect

modRNSPD2<-nls(Rt ~ b * (1 - (SPDt1/(k+d*Palm))), data=dat1, start=list(b=0.47, k=0.002, d=0.001), trace=TRUE)

### Extracting parameter values

summary(modRNSPD2)

# 3.- Fitting the logistic model with the reconstructed SOI (Yan et al. 2011) as a lateral perturbation effect

modRNSPD3<-nls(Rt ~ b * (1 - (SPDt1/(k+d*SOI))), data=dat1, start=list(b=0.47, k=0.002, d=0.001), trace=TRUE)

### Extracting parameter values

summary(modRNSPD3)

# 4.- Fitting the losgistic models using Palm cover and SOI as lateral perturbation effects

modRNSPD4<-nls(Rt ~ b * (1 - (SPDt1/(k+d*Palm+e*SOI))), data=dat1, start=list(b=0.47, k=0.002, d=0.001, e=-0.001), trace=TRUE)

### Extracting parameter values

summary(modRNSPD4)

### Extracting AIC values for all models

AIC(modRNSPD1, modRNSPD2, modRNSPD3, modRNSPD4)

### Extracting Log-likehood values for all models

logLik(modRNSPD1)

logLik(modRNSPD2)

logLik(modRNSPD3)

logLik(modRNSPD4)

### Calculating pseudo R2 for all models

cor(predict(modRNSPD1), dat1$Rt)^2

cor(predict(modRNSPD2), dat1$Rt)^2

cor(predict(modRNSPD3), dat1$Rt)^2

cor(predict(modRNSPD4), dat1$Rt)^2

**#Fitting linear regression models for the time series of pollen % of palm trees from Rapa Nui**

## Read the data

dat2<-read.table('rapnveget.txt',dec='.',sep='\t',header=TRUE)

## In the table Palms is % pollen of palm trees, SPD is the logarith of SPD value for the same date, and SOI is the logarithm of SOI index.

# 1.- Fitting a regression model using only SPD

modRNPALM1<-lm(Palms~(SPD), data=dat2)

### Extracting parameter values

summary(modRNPALM1)

# 2.- Fitting a regression model using only SOI

modRNPALM2<-lm(Palms~(SOI), data=dat2)

### Extracting parameter values

summary(modRNPALM2)

# 3.- Fitting a regression model using SOI and SPD without an interaction term

modRNPALM3<-lm(Palms~(SPD) + (SOI), data=dat2)

### Extracting parameter values

summary(modRNPALM3)

# 4.- Fitting a regression model using SOI and SPD with an interaction term

modRNPALM4<-lm(Palms~(SPD) + (SOI) + (SPD):(SOI), data=dat2)

### Extracting parameter values

summary(modRNPALM4)

# 5.- Fitting a regression model using SOI and an interaction term between SOI and SPD

modRNPALM5<-lm(Palms~(SPD):(SOI)+(SOI), data=dat2)

### Extracting parameter values

summary(modRNPALM5)

# 6.- Fitting a regression model using SPD and an interaction term between SOI and SPD

modRNPALM6<-lm(Palms~(SPD):(SOI)+(SPD), data=dat2)

### Extracting parameter values

summary(modRNPALM6)

### Extracting AIC values for all models

AIC(modRNPALM1,modRNPALM2, modRNPALM3, modRNPALM4, modRNPALM5, modRNPALM6)

### Extracting Log-likehood values for all models

logLik(modRNPALM1)

logLik(modRNPALM2)

logLik(modRNPALM3)

logLik(modRNPALM4)

logLik(modRNPALM5)

logLik(modRNPALM6)

#---------------------------------------------------------------------------------------------------

**# Author: Sergio A. Estay**

**# Date: 2003**

**# Institution: Center Of Applied Ecology and Sustainability (CAPES), PUC Chile**

**# Modified: Apr, 2017 by Jose T. Montero and Rodrigo Wiff**

**# Objective: this function makes a simulation of a population dynamic model, plots the simulation and its 95% CI bands based on either the "confint" fucntion over the model object or a "nlsBoot"**

**# Bootstrap, estimating then the upper and lower limit CI for the model parameter which are then used to make the CI bands.**

**# Instruction: The main change mad to the old version is the addition of the CI band and years x-axis in the main function. To add the years make a sequence, ussing the "c()" function withvthe desire start and end. For the CI band, the code is ready. You now just have to add**

#---------------------------------------------------------------------------------------------------

# Glosary of the function:

# No = inicial No value

# Mobj = model object from the nls output

# years = years of the simulation or original time series

# V1..x = predictor variables

simula2<-function(No,Mobj,years,Nt,V1, V2, V3){

# require libraries

require(nlstools)

require(MASS)

# Extract parameters from the modelo object

param <- c(Mobj$m$getAllPars())

set.seed(6)

pr <- mvrnorm(1000, param, vcov(Mobj))

dat1<-(RAPANUINW)

# Create object for output matrices and make the parametric sampling

p1 <- No

N2 <- matrix(NA, nrow = 22, ncol = 5000)

N3 <- matrix(NA, nrow = 22, ncol = 5000)

qqN3 <- matrix(NA, nrow = 22, ncol = 3)

qqN2 <- matrix(NA, nrow = 22, ncol = 3)

# Nested loop for CI and predictions based in the 5000 sampled parameters

for(t in 1:length(V1)){

N1a<-p1

N1b<-Nt[t]

for(j in 1:length(pr[,1])){

# model prediction with all generated parraneters N distributed

N2[t,j] <- N1a*exp(pr[j,1]*(1-(N1a/(pr[j,2]+pr[j,3]*V1[t]+pr[j,4]*V2[t]))))

# model prediction with all generated parraneters N distributed

N3[t,j] <- N1b*exp(pr[j,1]*(1-(N1b/(pr[j,2]+pr[j,3]*V1[t]+pr[j,4]*V2[t]))))

# CI estimation trough gettin the .25, .5 and .975

# quantiles of the normal distribution for each prediction (year)

qqN3[t,] <- quantile((N3[t,]),c(0.025,0.5,0.975), na.rm = T)

qqN2[t,] <- quantile((N2[t,]),c(0.025,0.5,0.975), na.rm = T)

p1<-qqN2[t,2]

}

}

na.pad <- function(x,len){

x[1:len]

}

makePaddedDataFrame <- function(l,...){

maxlen <- max(sapply(l,length))

data.frame(lapply(l,na.pad,len=maxlen),...)

}

# create data frame for de predictions and CI bands

out.est <- makePaddedDataFrame(list(years = years,

Nt = Nt,

Sim1 = qqN3[,2],

Sim2 = qqN2[,2],

Sim1.CI.L = qqN3[,1],

Sim1.CI.U = qqN3[,3],

Sim2.CI.L = qqN2[,1],

Sim2.CI.U = qqN2[,3]))

print(out.est)

# Plotting the Nt data

par(mar=c(4.5,5,2.5,5),lwd=1,bty="l",cex=0.7)

plot(out.est[,2]~out.est[,1], xlab="Years AD", ylab="", cex=1.4,

cex.lab=1, cex.axis=1, tck=0.02,

type="p",

pch=16,

ylim=c(0, 0.0028),

#ylim=c(min(out.est[,2],na.rm=TRUE)-1,1.1*max(out.est[,2],na.rm=TRUE)),

xlim=c(min(out.est[,1],na.rm=TRUE),max(out.est[,1],na.rm=TRUE)))

text(1750, 0.0026, "D", cex=1.5)

#rect(2004, 10, 2016, 1500, col="grey75", density=85, border=NA, angle=45, lty=1)

#rect(1975, 10, 1984, 1500, col="grey75", density=85, border=NA, angle=45, lty=1)

# Ploting prediction and CI curves

points((out.est[,2])~out.est[,1], type="p", col="black", pch=16)

# N2 prediction

points((out.est[,4])~out.est[,1],col="red",type="l", lwd=4, lty=1)

# N3 prediction

#points(out.est[,3]~out.est[,1],col="blue", type="l", lty=1, lwd=2)

# Poligon for the 95% CI region

# CI Simulation I

#polygon(c(out.est[,1],rev(out.est[,1])),

#c(out.est$Sim1.CI.U, rev(out.est$Sim1.CI.L)),

#col=rgb(.224, .224, .224,0.3), border=NA)

# CI Simulation II

polygon(c(out.est[,1],rev(out.est[,1])),

c((out.est$Sim2.CI.U), rev((out.est$Sim2.CI.L))),

col=rgb(.224, .224, .224,0.3), border=NA)

return(out.est)

}

#simula2<-function(No,Mobj,years,Nt,V1,V2,V3){

simm1<-simula2(0.000077,modRNSPD4,seq(1130,1760, 30), RAPANUINW$SPDsm2, RAPANUINW$PalmRR, RAPANUINW$SOI1st)

# Example of how to use the function:

# sim.m1 <- simula(98, m1 , c(1993:2016), lng$LCS, lng$ELCS)

param <- c(modRNSPD4$m$getAllPars())

predR2[1] <- 1 - (sum((simm1$Sim2 - simm1$Nt)^2)/sum((simm1$Nt - mean(simm1$Nt))^2))
